# Reduced tolerogenic factor sCD83 in NMOSD and relapsing MOGAD: a potential new therapeutic pathway

**DOI:** 10.3389/fimmu.2025.1620069

**Published:** 2025-07-24

**Authors:** Ariel Rechtman, Omri Zveik, Lyne Shweiki, Garrick Hoichman, Tal Friedman-Korn, Livnat Brill, Adi Vaknin-Dembinsky

**Affiliations:** ^1^ Department of Neurology and Laboratory of Neuroimmunology and the Agnes-Ginges Center for Neurogenetics, Hadassah-Hebrew University Medical Center, Ein–Kerem, Jerusalem, Israel; ^2^ Faculty of Medicine, Hebrew University of Jerusalem, Jerusalem, Israel; ^3^ Department of Military Medicine, Faculty of Medicine, Hebrew University of Jerusalem, Jerusalem, Israel

**Keywords:** MOGAD, NMOSD, demyelinating CNS disease, CD83, soluble CD83, azathioprine, IVIg, mycophenolate mofetil

## Abstract

**Background:**

Neuromyelitis optica spectrum disorders (NMOSD) and myelin oligodendrocyte glycoprotein antibody-associated disease (MOGAD) are autoimmune central nervous system disorders with poorly understood immune pathways. CD83 plays a crucial role in the development and maintenance of immune tolerance. This study aims to evaluate CD83 expression in NMOSD and MOGAD and its correlation with disease activity.

**Methods:**

RNA extracted from PBMCs of MOGAD, NMOSD and healthy controls (HCs) was analyzed to assess CD83 expression levels. ELISA was used to quantify soluble CD83 (sCD83) levels in the CSF and serum of patients. Additionally, the effects of therapeutic agents used for CNS demyelinating diseases on sCD83 expression levels were examined. The study enrolled 231 untreated participants, including 64 with MOGAD, 56 with NMOSD, 47 with MS, and 64 HCs.

**Results:**

NMOSD patients exhibited lower sCD83 levels compared to MOGAD and HCs, and MOGAD patients with a relapsing course had lower sCD83 levels than those with a monophasic course. Lower sCD83 levels correlated with a severe disease course. Treatment with IVIG and azathioprine significantly increased sCD83 levels in the patients’ serum. *In vitro* treatment with immunosuppressives led to a significant increase in sCD83 levels with the most pronounced effect observed following treatment with mycophenolate mofetil.

**Discussion:**

Our study consistently found lower sCD83 levels in NMOSD and relapsing MOGAD patients. sCD83 levels increased following IVIG and immunosuppressive therapy. This elevation may reflect either a direct effect of the therapy itself, or a compensatory rebound response following immune suppression. These findings highlight the potential of sCD83 as a prognostic biomarker in these diseases and support its role as both a therapeutic target and a marker for treatment response in CNS demyelinating disorders.

## 1 Introduction

Neuromyelitis optica spectrum disorders (NMOSD) and myelin oligodendrocyte glycoprotein (MOG) antibody-associated disease (MOGAD) are autoimmune inflammatory disorders of the central nervous system (CNS) (1). NMOSD and MOGAD have a relapsing course, with heterogeneous recovery between episodes (1). NMOSD typically involves several relapses, whereas MOGAD may occur either as a single episode or with recurring episodes, and generally, MOGAD has a more favorable outcome (1).

NMOSD and MOGAD exhibit distinct immunopathological processes (2). NMOSD is primarily driven by autoantibodies against aquaporin-4 (AQP4), leading to astrocyte damage and secondary demyelination, characterized by complement-mediated cytotoxicity and severe inflammation (3). In contrast, MOGAD involves an autoimmune response against the MOG protein expressed on oligodendrocytes, characterized by inflammatory demyelination, perivenous demyelination, and predominance of CD4+ T-cell infiltration (1).

Adequate and early treatment of relapses in NMOSD and MOGAD is crucial, considering the high probability of accumulating residual impairment from relapse, resulting in significant motor and visual disability especially in NMOSD patients (2). While most individuals with MOGAD show good response to acute treatments, some patients with NMOSD may not respond as effectively (2).

Rituximab, mycophenolate mofetil (MMF), methotrexate, and azathioprine (AZA) are traditional medications used to prevent relapses in NMOSD patients (4). In addition, three disease-modifying therapies have been approved by the FDA specifically for NMOSD treatment: eculizumab, inebilizumab, and satralizumab. These drugs have shown high effectiveness in reducing the frequency of relapses in NMOSD (5, 6). In the PREVENT trial, eculizumab reduced relapse risk by 94%, with 88% of patients remaining relapse-free over 3.7 years (7). Inebilizumab reduced relapse risk by 73% in the N-MOmentum trial, with 77% relapse-free at four years (8). Satralizumab, evaluated in the SAkuraSky and SAkuraStar trials, reduced relapse risk by 79% in AQP4-IgG–positive patients, with 89% relapse-free at 48 weeks (9, 10).

Currently, no medications have received FDA approval specifically for MOGAD treatment, but clinical trials are underway for several drugs, including satralizumab and rozanolixizumab. Since relapsing MOGAD patients require long-term treatment, physicians often prescribe FDA-approved medications for other conditions, such as intravenous immunoglobulin (IVIG), AZA, MMF, rituximab, and tocilizumab (11).

Research on new therapeutic strategies for NMOSD is currently centered on restoring immune tolerance (12). The development and implementation of therapies based on immune tolerance in NMOSD is likely to represent crucial progress toward improving treatment outcomes for the disease. Immune tolerance involves the prolonged or permanent modulation of aberrant immune responses toward a homeostatic state, critical for maintaining immune system balance (13). This process occurs through both central and peripheral immune mechanisms (14, 15), and is known to play a pivotal role in various autoimmune diseases including CNS demyelinating diseases (16). For example thymic B cells contribute to tolerance against a group of germinal center-associated antigens, such as AQP-4 (17). Notably, defects in B cell tolerance are linked with the onset and progression of NMOSD (18). These defects typically lead to increased prevalence of systemic autoantibodies and heightened B cell activation (18).

The CD83 molecule plays a crucial role in the development and maintenance of immune tolerance (19, 20). It is expressed on various activated immune cells, including B and T lymphocytes, monocytes, dendritic cells (DCs), microglia, and neutrophils (19). CD83 is essential for modulating and resolving immune responses and has been shown to regulate cellular activation, contributing to the resolution of neuroinflammation in microglial cells (20).

CD83 has two forms: a membrane-bound (mCD83) and a soluble form (sCD83) (21). mCD83 is expressed on activated B and T lymphocytes and DCs and is widely considered a marker of cellular activation in antigen presenting cells (19). It plays a crucial role in immune regulation by promoting maturation, activation, and maintenance of immune homeostasis (19). Conversely, sCD83 exhibits immunomodulatory capabilities, notably inducing responses from tolerogenic DCs and regulatory T (Treg) cells (22, 23). sCD83 interacts with the TLR4/MD-2 receptor complex, leading to inhibition of T cell proliferation and the induction of anti-inflammatory mediators by activating the TGFB/indoleamine-2,3-dioxygenase axis (24). sCD83 secreted by CD83+ B cells reduced, interleukin (IL)-1β, IL-18, and interferon (IFN)-γ secretion by DCs (25).

Studies have shown that knockout of CD83 in Tregs results in a more highly activated, pro-inflammatory phenotype (26) and that CD83 knockout in the autoimmune animal models, EAE and colitis, consistently demonstrated a more severe disease course (27, 28). Additionally, CD83 knockout in microglia leads to an over-activated state during neuroinflammation, emphasizing its role as a regulator of the CNS immune response (20).

Given its tolerogenic properties, sCD83 is emerging as a promising therapeutic candidate for demyelinating diseases. sCD83 has demonstrated a potent therapeutic effect in EAE, uveitis and allergic rhinitis models (29–31). In EAE, sCD83 effectively reduces disease severity by inhibiting DC maturation, altering DC-T cell interactions, and promoting Treg activity (29). This leads to reduced T cell proliferation and inflammatory cytokine production, resulting in significant attenuation of paralysis and enhanced recovery (29). Similarly, in the uveitis model, sCD83 alleviates inflammation by modulating filamentous actin-dependent calcium signaling in DCs, thereby reducing T cell activation and inflammatory cytokine expression (31).

The counterparts of macrophages and DCs as antigen-presenting cells in the CNS are microglia (32). CD83+ microglia have recently been shown to be vital in maintaining CNS homeostasis and modulating neuroinflammation. Dysregulation of these cells has been linked to the progression of neurodegenerative diseases. In EAE, CD83 expression in microglia promotes the resolution of inflammation by limiting the influx of pathogenic immune cells to the CNS (20). In Alzheimer’s disease (AD), a specific CD83+ microglial subtype has been identified in 47% of patients, and is associated with protective immune responses, including antigen presentation and amyloid clearance (33, 34). However, these cells are reduced in AD, contributing to disease progression (33, 34). Similarly, in Parkinson’s disease, the loss of CD83+ microglia in affected brain regions is linked to increased neuroinflammation and neuronal vulnerability (35). Additionally, sCD83 induces an anti-inflammatory macrophage phenotype by upregulating inhibitory markers like ILT-2 and CD163 and downregulating activation markers such as MHC-II (36). Beyond its role in resolving immune responses, sCD83 is vital in the differentiation of T and B lymphocytes and in sustaining immunological tolerance (22, 37).

Studies on sCD83 in NMOSD and MOGAD have not yet been conducted. However, the promising results of sCD83 treatment in EAE and the severe disease course of EAE animals lacking CD83, combined with the lack of data on CD83 in NMOSD and MOGAD, highlights the need for further investigation of CD83 in CNS demyelinating diseases. The current study aims to examine CD83 expression in these conditions and correlate the findings with disease activity.

## 2 Methods

### 2.1 Ethics

The study was approved by Hadassah Medical Organization Ethics Committee (HMO-08-0589) All the participants provided written informed consent.

### 2.2 Patients

231 individuals participated in this study, including 64 MOGAD patients, 56 NMOSD patients, 47 MS patients, and 64 healthy controls (HCs). MOGAD patients were diagnosed using the international diagnostic criteria (38, 39), MS patients were diagnosed according to the 2017 McDonald criteria, and NMOSD patients met the 2015 diagnostic criteria (40, 41). A serum sample was available for all patients, whereas peripheral blood mononuclear cells (PBMCs) and cerebrospinal fluid (CSF) were available for (37 MOGAD, 20 NMOSD, 17 MS, and 40 HCs) and (37 MOGAD, 26 NMOSD, 23 MS, and 15 ONNIDs), respectively.

All patients with MOGAD and NMOSD who visited the neurology clinic or department at Hadassah Ein Karem Hospital and agreed to participate were included in the study. Pregnant patients, those currently receiving immunosuppressive treatment, and those who were treated with steroids for less than three months before the blood sample, were excluded from the disease group analysis. Clinical information such as age, sex, disease duration, oligoclonal bands (OCB) status, expanded disability status scale (EDSS), and brain magnetic resonance imaging (MRI) was extracted from the patient’s files. The groups were comparable in terms of age and sex distribution (**Table 1**). Serum samples were obtained from 33 MOGAD patients in remission and 31 during relapse, 26 NMOSD patients in remission and 30 during relapse, and 22 MS patients in remission and 25 during relapse.

**Table 1:** Clinical and demographic information of all participants.

Variables	HC (n=64)	MOGAD (n=64)	MS (n=47)	NMOSD (n=56)	p value
Age (years): mean ± std	33.22 ± 10.83	32.27 ± 13.36	34.66 ± 11.35	34.10 ± 12.19	0.73
Gender (F:M)	39:25	41:23	34:13	44:12	0.16
EDSS		1.45 ± 1.38	3.03 ± 2.48	3.62 ± 2.26	<0.0001
Disease Duration (years): mean ± std		2.33 ± 4.64	2.59 ± 2.66	2.28 ± 3.34	0.91
Oligoclonal Band (Negative: Positive)		42:9	12:30	40:12	<0.0001

HC, healthy control; MOGAD, myelin oligodendrocyte glycoprotein antibody-associated disease; NMOSD, Neuromyelitis optica spectrum disorders; MS, multiple sclerosis; EDSS, expanded disability status scale

MOGAD may present with a monophasic or relapsing disease course (1). For the purpose of this study, patients were classified as monophasic or relapsing based on their clinical course. Patients with less than two years of follow-up were excluded from this classification (**Table 2**).

**Table 2:** Clinical and demographic information of monophasic and relapsing MOGAD patients.

Variables	Monophasic MOGAD patients (n=21)	Relapsing MOGAD patients * (n=27)	p value
Age (years): mean ± std	30.37 ± 16.22	32.47 ± 10.92	0.60
Gender (F:M)	13:8	19:8	0.54
EDSS	1.29 ± 1.71	1.64 ± 1.14	0.38
Disease Duration (years): mean ± std	1.54 ± 2.63	2.86 ± 5.07	0.28
Oligoclonal Band (Negative: Positive)	16:2	15:6	0.18

* Patients were classified as having a monophasic course after at least 2.5 years of follow-up without relapse.

MOGAD, myelin oligodendrocyte glycoprotein antibody-associated disease; EDSS, expanded disability status scale

### 2.3 RNA isolation

PBMCs were isolated from freshly drawn heparinized blood utilizing Ficoll-Paque gradient centrifugation. Following isolation, the PBMCs were immediately suspended in 1 mL of TRI Reagent^®^ (Sigma-Aldrich) and stored at −80°C to preserve RNA integrity. RNA extraction was subsequently performed according to the manufacturer’s instructions.

### 2.4 Nanostring

The expression of specific gene transcripts was analyzed using the nCounter Immunology Panel (Nanostring Technologies, Seattle, WA, USA) following the manufacturer’s protocol. Data analysis was performed with the nSolver analysis software (Nanostring Technologies).

### 2.5 Bioinformatics analysis

Differently expressed genes are presented as a scatter plot, y-axis: log2 non-adjusted p-value for monophasic and relapsing MOGAD patients, x-axis: log2 fold change (Monophasic/Relapsing) The Database for Annotation, Visualization, and Integrated Discovery was used to study shared biological pathways of significant differentially expressed genes.

### 2.6 RT-PCR

cDNA was produced from 250 ng total RNA with a qScript cDNA Synthesis Kit (Quanta Biosciences, Gaithersburg, MD, USA), according to the manufacturer’s instructions. Quantitative polymerase chain reaction (PCR) was performed using PerfeCTa SYBR Green FastMix Rox (Quanta Biosciences). Gene amplification was carried out using the StepOnePlus real-time (RT) PCR system (Applied Biosystems). The threshold cycle value (2 − ΔCT) was used for statistical analysis. All target mRNAs were normalized to the hypoxanthine-guanine phosphoribosyltransferase (HPRT) reference gene. Expression of each gene was evaluated in triplicate. Primers used (Agentek):

HPRT F: 5’ CTGGCAAAACAATGCAGACTTT R: 5’ GGTCCTTTTCACCAGCAAGCT

sCD83 F: 5’ CTGTAAGGCACATGGAGGTGA R: ATTGCCAGCTTTGTAAAGCCATT

### 2.7 sCD83 Enzyme-linked immunosorbent assay

Levels of sCD83 were measured across various biological samples in duplicates, including sera (4x dilution), CSF (without dilution), and supernatants of cultured PBMCs obtained from the study participants (without dilution). These measurements were performed using the Human sCD83 Quantikine HS ELISA Kit (DY2044-05, Range 39.1 - 2,500 pg/mL, R&D Systems, Bio-Techne, Abingdon, UK).

### 2.8 PBMCs cultured with different drugs

PBMCs were isolated from freshly drawn heparinized blood using Ficoll-Paque gradient centrifugation. Once isolated, PBMCs were incubated in a 24-well dish at a density of 2×10^6 cells per 1 mL, and then they were treated with various drugs for 48 hours without replacing the media. The concentration of each drug was determined based on a review of previous *in vitro* studies (**Supplementary Table 1**). We tested drugs commonly used to treat demyelinating disorders.

The effect of the drug treatments on sCD83 secretion was quantified by calculating the fold change, expressed as the log2 of the ratio of post-treatment to pre-treatment concentration levels of sCD83. Drugs were categorized based on their impact on sCD83 secretion: a log2 fold change greater than 0.6 with a p-value less than 0.01 following multiple comparison correction was considered a significant increase, while a log2 fold change less than -0.6 with a p-value less than 0.01 indicated a significant decrease.

### 2.9 Brain volume and VEP analysis

T1-weighted images, were performed using MRI scanners at Hadassah Ein Kerem Medical Center, as detailed previously (42). The volumetric analysis of these images was conducted using the MDBrain platform (42). This advanced platform utilizes artificial intelligence to perform volumetric analysis of the brain, quantifying volume measurements and identifying deviations. It achieves this by comparing the scanned volumetric data against a standard database of normal brain volumes (42). Visual Evoked Potentials (VEP) results were obtained as described previously (43). In cases of bilateral optic neuritis, both affected eyes were included in the analysis.

### 2.10 Statistical analysis

We performed an analysis of variance (ANOVA) to compare sCD83 expression levels, and levels in serum and CSF results across the four groups, followed by Tukey’s *post-hoc* test to identify pairwise differences between groups, with correction for multiple comparisons. A Wilcoxon signed-rank test was used to assess differences in sCD83 levels before and after treatment in sera and PBMCs samples of patients. Correlations between sCD83 levels and both clinical and volumetric data were evaluated using Pearson’s correlation coefficient. Additionally, receiver operating characteristic (ROC) analysis was conducted to determine the area under the curve (AUC), assessing the utility of sCD83 levels as a predictor of relapsing disease. Statistical significance was defined as p < 0.05 for all analyses.

## 3 Results

### 3.1 Patients with NMOSD and relapsing MOGAD have lower sCD83 expression

#### 3.1.1 sCD83 levels are differently expressed in CNS demyelinating diseases

**Figure 1:**
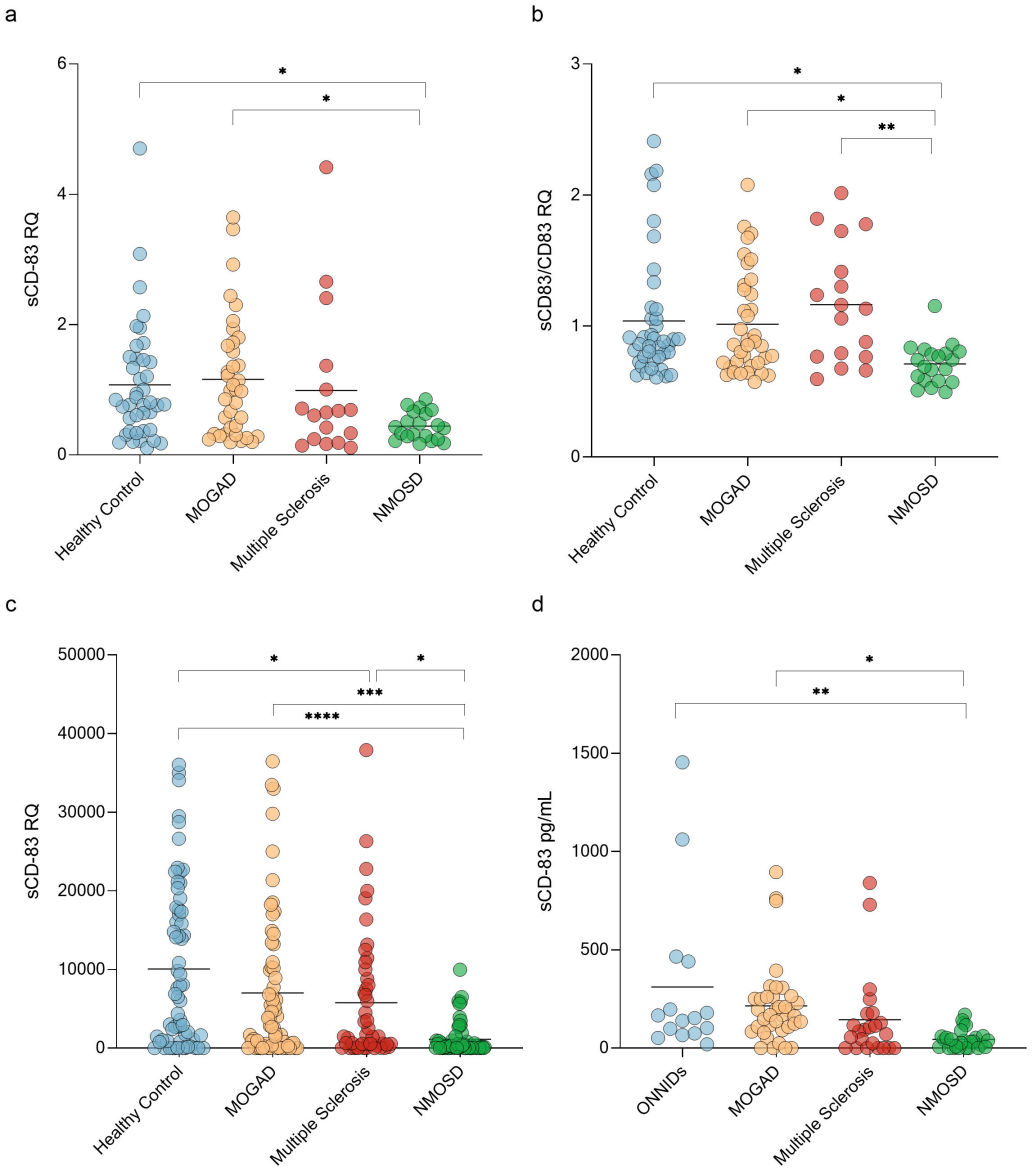
NMOSD patients have low sCD83 levels. **(a)** sCD83 expression in PBMCs of HCs (n=40), MOGAD (n=37), MS (n=17) and NMOSD patients (n=20). The expression level of NMOSD patients was significantly lower than that of MOGAD patients (0.44 ± 0.22 RQ vs 1.16 ± 0.92 RQ, p = 0.02), and HCs (0.44 ± 0.22 RQ vs 1.07 ± 0.91 RQ, p = 0.05) **(b)** sCD83/mCD83 expression ratio in PBMCs of HCs (n=40), MOGAD (n=37), MS (n=17) and NMOSD patients (n=20). The sCD83/mCD83 ratio of NMOSD patients was significantly lower than that of MOGAD patients (0.71 ± 0.16 vs 1.02 ± 0.39, p = 0.04), MS patients (0.71 ± 0.16 vs 1.16 ± 0.45, p=0.006) and HCs (0.71 ± 0.16 vs 1.04 ± 0.48, p = 0.02) **(c)** sCD83 levels in the sera of HCs (n=64), MOGAD (n=64), MS (n=47) and NMOSD (n=56) patients. NMOSD patients had significantly lower sCD83 levels compared to HCs (1129.34 ± 2073.49 pg/mL vs 10076.75 ± 10366.37 pg/mL, p<0.0001) and MOGAD (1129.34 ± 2073.49 pg/mL vs 7019.97 ± 9225.68 pg/mL, p=0.0008) patients. MS patients had significantly lower sCD83 concentration compared to HCs (5777.94 ± 8319.23 pg/mL vs 10076.49 ± 10366.21 pg/mL, p = 0.04) and higher sCD83 concentration compared to NMOSD patients (5777.94 ± 8319.23 pg/mL vs 1129.34 ± 2073.49 pg/mL, p = 0.03). **(d)** sCD83 levels in the CSF of ONNIDs (n=15), MOGAD (n=37), MS (n=23) and NMOSD (n=26) patients. NMOSD patients had significantly lower sCD83 levels compared to MOGAD patients (215.20 ± 202.80 pg/mL vs 44.77 ± 47.84 pg/mL, p=0.02) and ONNIDs (311.30 ± 411.80 pg/mL vs 44.77 ± 47.84 pg/mL, p=0.002). HC, healthy control; MOGAD, myelin oligodendrocyte glycoprotein antibody-associated disease; NMOSD, neuromyelitis optica spectrum disorders; MS, multiple sclerosis; ONNIDs, other non-inflammatory neurological disorders; sCD83, soluble CD83; PBMCs, Peripheral blood mononuclear cells. * p<0.05 ** p<0.01 *** p<0.001 **** p<0.0001.

A key immunopathological process in CNS demyelinating diseases involves defects in immune tolerance mechanisms (44). Given that sCD83 plays a critical role in maintaining immune tolerance, we studied its expression in these disorders. We first analyzed the expression of sCD83 in PBMCs from untreated patients with MOGAD, MS, and NMOSD using RT-PCR (with specific primers designed to detect the soluble form of CD83). sCD83 expression was significantly different between the groups (ANOVA; p=0.03). sCD83 levels were lower in NMOSD patients compared to MOGAD patients (0.44 ± 0.22 RQ vs 1.16 ± 0.92 RQ, p = 0.02, **Figure 1a**), and HCs (0.44 ± 0.22 RQ vs 1.07 ± 0.91 RQ, p = 0.05, **Figure 1a**). No significant difference was observed between MOGAD patients, MS patients and HCs. (**Figure 1a**). We then analyzed the sCD83/CD83 expression ratio across all groups and found significant differences (ANOVA, p = 0.006). NMOSD patients exhibited a lower sCD83/CD83 expression ratio compared to HCs (0.71 ± 0.16 vs 1.04 ± 0.48, p = 0.02, **Figure 1b**), MOGAD patients (0.71 ± 0.16 vs 1.02 ± 0.39, p = 0.04, **Figure 1b**), and MS patients (0.71 ± 0.16 vs 1.16 ± 0.45, p=0.006, **Figure 1b**). No significant difference was found between the MOGAD, MS and HCs groups. (**Figure 1b**)

Since sCD83 can be generated through two distinct mechanisms—shedding of mCD83 or alternative splicing—it is important to assess sCD83 protein levels alongside mRNA expression. To measure sCD83 concentrations in the sera and CSF of these patients, we used an ELISA assay. In the sera, a significant difference was observed between the four groups (ANOVA; p < 0.0001). Specifically, sCD83 expression was significantly lower in both NMOSD (1129.34 ± 2073.49 pg/mL vs 10076.49 ± 10366.21 pg/mL, p < 0.0001, **Figure 1c**) and MS patients (5777.94 ± 8319.23 pg/mL vs 10076.49 ± 10366.21 pg/mL, p = 0.04, **Figure 1c**) when compared to HCs. Furthermore, sCD83 levels in NMOSD patients were significantly lower than in MOGAD (1129.34 ± 2073.49 pg/mL vs 7019.97 ± 9225.68 pg/mL, p = 0.0008, **Figure 1c**) and MS patients (1129.34 ± 2073.49 pg/mL vs 5777.94 ± 8319.23 pg/mL, p = 0.03, **Figure 1c**). No significant differences were found when comparing MOGAD to MS (7019.97 ± 9225.68 pg/mL vs 5777.94 ± 8319.23 pg/mL, p = 0.86, **Figure 1c**) or to HCs (7019.97 ± 9225.68 pg/mL vs 10076.49 ± 10366.21 pg/mL, p = 0.16, **Figure 1c**).

Further analysis of sCD83 levels in the CSF showed significantly differences between the 4 groups (ANOVA; p=0.002). NMOSD patients had significantly lower sCD83 levels compared to MOGAD patients (215.20 ± 202.80 pg/mL vs 44.77 ± 47.84 pg/mL, p=0.02, **Figure 1d**) and other non-inflammatory neurological disorders (ONNIDs) (311.30 ± 411.80 pg/mL vs 44.77 ± 47.84 pg/mL, p=0.002, **Figure 1d**). Additionally, no significant differences were observed between MS and MOGAD (144.00 ± 219.20 pg/mL vs 215.20 ± 202.80 pg/mL, p = 0.64, **Figure 1d**), NMOSD (144.00 ± 219.20 pg/mL vs 44.77 ± 47.84 pg/mL, p = 0.42, **Figure 1d**), or ONNID patients (144.00 ± 219.20 pg/mL vs 67.71 ± 104.20 pg/mL, p = 0.12, **Figure 1d**).

#### 3.1.2 sCD83 levels are lower in the sera of patients with relapsing MOGAD

One measure of severity in MOGAD is whether a patient experiences a monophasic form or a more active relapsing form. We therefore compared sCD83 expression and concentration in serum and CSF between MOGAD patients with monophasic versus relapsing disease.

**Figure 2:**
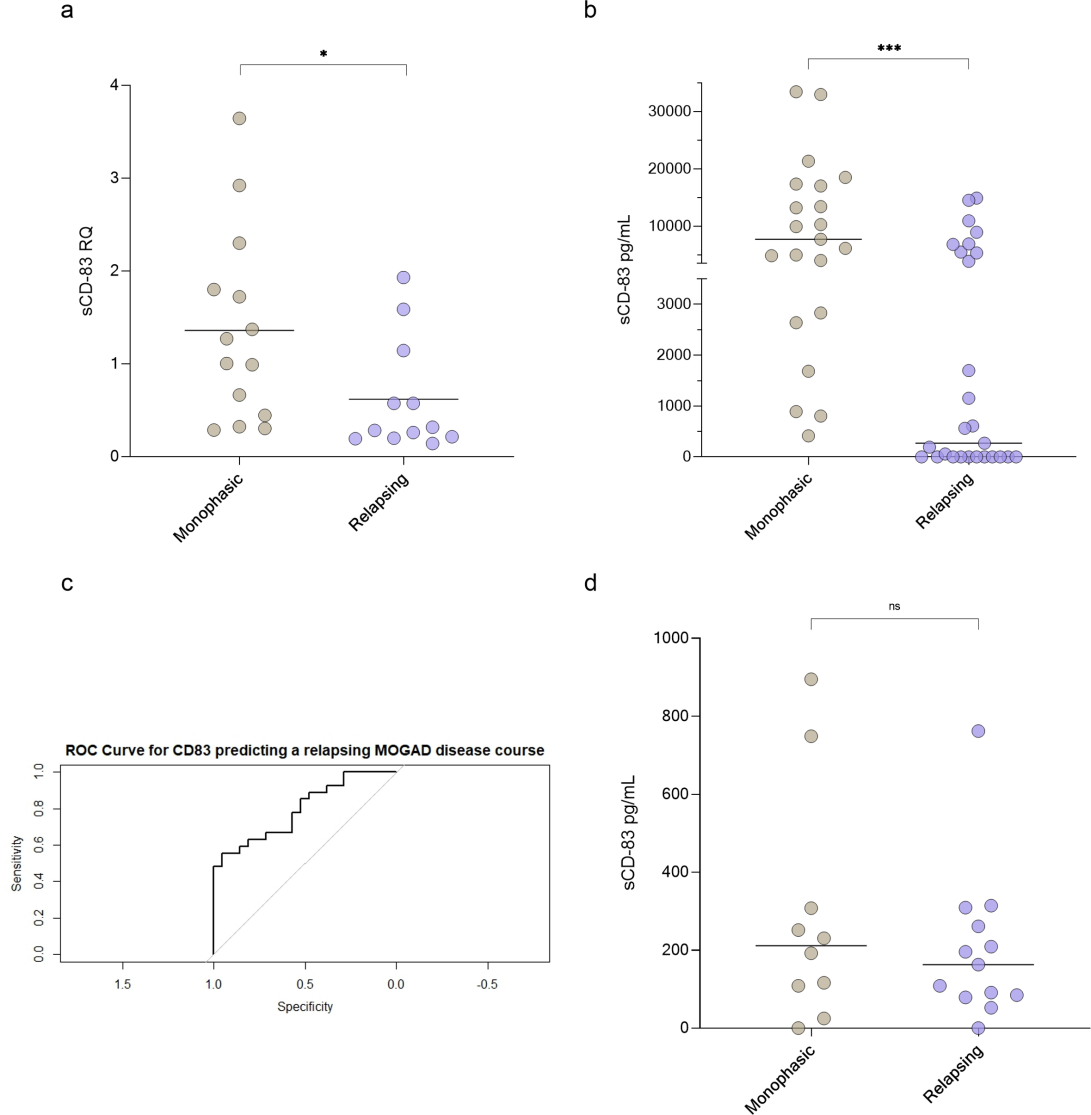
sCD83 levels can predict a relapsing MOGAD disease course. **(a)** sCD83 expression of monophasic (n=14) versus relapsing (n=12) MOGAD patients (1.36 ± 1.03 RQ vs 0.62 ± 0.60 RQ, p=0.04). **(b)** sCD83 levels in the sera of monophasic (n=21) and relapsing (n=27) MOGAD patients. Monophasic MOGAD patients had significantly higher sCD83 levels compared to relapsing MOGAD patients (10694.80 ± 9846.70 pg/mL vs 3049.57 ± 4621.34 pg/mL, p=0.0009) **(c)** For sCD83 in the sera, a value of 706.85 pg/mL yielded the highest ROC-AUC of 0.811 (with a sensitivity of 59.26% and specificity of 95.24% **(d)** sCD83 levels in the CSF of monophasic (n=10) and relapsing (n=13) MOGAD patients. No significant difference was found between monophasic and relapsing MOGAD patients (287.60 ± 299.80 pg/mL vs 202.30 ± 194.90 pg/mL, p = 0.42). MOGAD: myelin oligodendrocyte glycoprotein antibody-associated disease; sCD83: soluble CD83; ROC: receiver operating characteristic; AUC: area under the curve. * p<0.05 ** p<0.01 *** p<0.001 **** p<0.0001.

Using RT-PCR, we found that sCD83 expression was significantly lower in relapsing MOGAD patients compared to those with monophasic disease (0.62 ± 0.60 RQ vs 1.36 ± 1.03 RQ, p = 0.04, **Figure 2a**). Similarly, sCD83 levels in the sera were significantly lower in relapsing patients than in those with the monophasic form (3049.57 ± 4621.34 pg/mL vs 10694.80 ± 9846.70 pg/mL, p = 0.0009, **Figure 2b**). To further distinguish between monophasic and relapsing MOGAD patients, we conducted a ROC analysis. A serum sCD83 concentration of 706.85 pg/mL was identified as the most specific threshold for predicting a relapsing disease course, with specificity of 95.24%, sensitivity of 59.26%, and an AUC of 0.811 (**Figure 2c**). In contrast to the significant findings in serum, no significant difference was observed in CSF between relapsing and monophasic MOGAD patients (202.30 ± 194.90 pg/mL vs 287.60 ± 299.80 pg/mL, p = 0.42, **Figure 2d**).

To further validate these findings, we re-analyzed a previously conducted immunological Nanostring gene array in MOGAD patients (45). This analysis revealed that CD83 expression was markedly higher in monophasic MOGAD compared to the relapsing form (3219.75 ± 1814.36 vs 295.01 ± 184.97), supporting the differential expression of CD83 across disease phenotypes.

For 19 MOGAD, 7 NMOSD patients and 6 MS patients we measure sCD83 levels during both relapse and remission. For all groups, a slightly non-significant upregulation was seen during relapse (MOGAD: 4940.14 ± 6155.12 vs 6803.20 ± 8977.60, p= 0.18, **Supplementary Figure S1a**; NMOSD: 88.86 ± 185.50 vs 348.30 ± 779.80, p=0.29, **Supplementary Figure S1b**; MS: 3427.21 ± 5236.38 vs 5547.27 ± 6779.68, p=0.53, **Supplementary Figure S1c**).

Additionally, sCD83 levels did not differ significantly between relapse and remission samples when analyzed separately for each disorder (MOGAD: 7119.32 ± 10232.09 vs 6913.49 ± 8186.14, p=0.93, **Supplementary Figure S2a**; NMOSD 1066.75 ± 1831.68 vs 1183.35 ± 2292.41, p=0.84, **Supplementary Figure S2b**; MS 5989.19 ± 9332.90 vs 5590.15 ± 7508.02, p=0.87, **Supplementary Figure S2c**).

#### 3.1.3 Lower sCD83 levels associated with reduced brain volume and prolonged VEP

**Figure 3:**
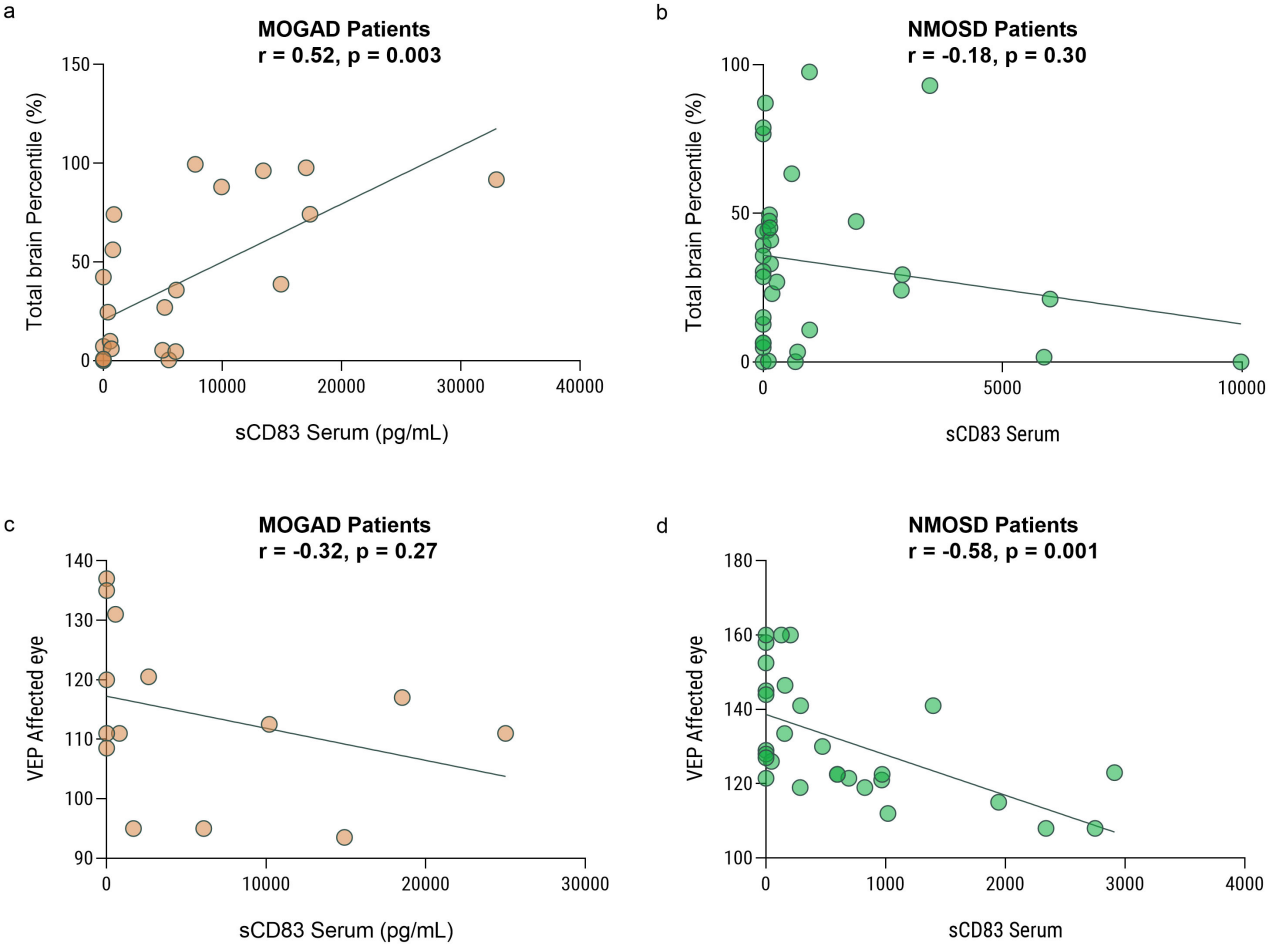
Low sCD83 levels correlate with a severe disease course in MOGAD and NMOSD patients. Correlation between sCD83 levels and the volume of the brain and VEP in MOGAD and NMOSD patients. **(a)** There is a significant correlation between total brain volume and sCD83 level in the sera of MOGAD patients (r = 0.66, p = 0.0004) **(b)** There is no significant correlation between total brain volume and sCD83 level in the sera of NMOSD patients (r = -0.28, p = 0.10) **(c)** There is no significant correlation between VEP and sCD83 level in the sera of MOGAD patients (r=-0.26, p=0.35). **(d)** There is a significant correlation between VEP and sCD83 level in the sera of NMOSD patients (r = -0.50, p = 0.008) MOGAD, myelin oligodendrocyte glycoprotein antibody-associated disease; NMOSD, Neuromyelitis optica spectrum disorders; sCD83, soluble CD83; VEP, Visual Evoked Potentials.

We further investigated the association between paraclinical disease severity measures and sCD83 levels in both MOGAD and NMOSD patients. We analyzed high-quality brain images from 24 and 35 individuals with MOGAD and NMOSD, respectively. We found a significant positive correlation between serum sCD83 levels and normalized total brain volume of MOGAD patients (r = 0.66, p = 0.0004, **Figure 3a**). No correlation was observed between sCD83 levels and brain volume of NMOSD patients (r = -0.28, p = 0.10, **Figure 3b**).

We then analyzed the VEP score in the affected eyes of 15 MOGAD and 27 NMOSD patients with ON. We found a significant negative correlation between serum sCD83 levels and the VEP score in NMOSD patients (r = -0.50, p = 0.008, **Figure 3c**). No significant correlation between sCD83 levels and the VEP of MOGAD patients was found (r=-0.26, p=0.35, **Figure 3d**). Furthermore, we performed a correlation between sCD83 levels and EDSS scores in both MOGAD (r=-0.13, p=0.35), MS (r=-0.20, p=0.20) and NMOSD patients (r=0.07, p=0.73) and found no significant associations.

### 3.2 sCD83 levels are influenced by treatments administered to patients with CNS demyelinating diseases

#### 3.2.1 Increased sCD83 levels following IVIG treatment in the sera of MOGAD and NMOSD patients

**Figure 4:**
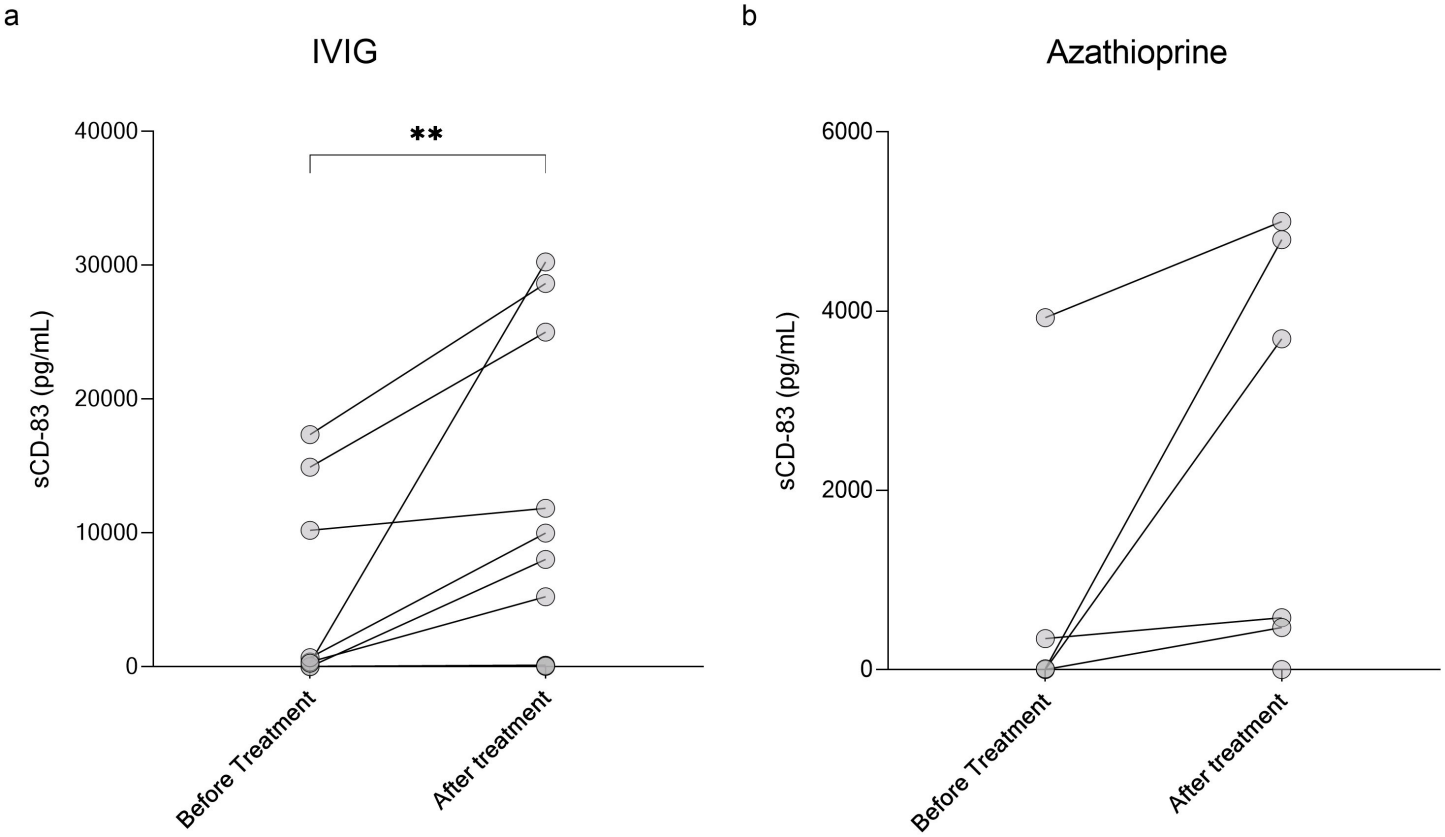
Elevation of sCD83 levels in serum following IVIG treatment in MOGAD and NMOSD patients. Serum levels of sCD83 in MOGAD and NMOSD patients before and after initiating treatment. **(a)** Changes in serum sCD83 levels in MOGAD and NMOSD patients before and after treatment with IVIG (n=12, from 3650.21 ± 6523.94 pg/mL to 9937.99 ± 11710.02 pg/mL, p=0.002). **(b)** Changes in serum sCD83 levels in MOGAD and NMOSD patients before and after treatment with Azathioprine (n=6, from 714.10 ± 1580.23 pg/mL to 2423.23 ± 2323.79 pg/mL, p=0.06). MOGAD, myelin oligodendrocyte glycoprotein antibody-associated disease; NMOSD, neuromyelitis optica spectrum disorders; sCD83, soluble CD83; IVIG, intravenous immunoglobulin. * p<0.05 ** p<0.01 *** p<0.001 **** p<0.0001.

Given its role in immune tolerance, sCD83 may have therapeutic potential for CNS demyelinating diseases. To explore this, we first analyzed the impact of various treatments on sCD83 levels in the serum of patients. We measured serum sCD83 levels in patients before and after initiating treatment with methotrexate, IVIG, natalizumab, rituximab, ocrelizumab, AZA, and cladribine. The results for all treatments are detailed in **Table 3**. Notably, IVIG significantly increased sCD83 concentrations in the serum of MOGAD and NMOSD patients (n=12, from 3650.21 ± 6523.94 pg/mL to 9937.99 ± 11710.02 pg/mL, p=0.002, **Figure 4a**), with a similar upward trend observed for AZA (n=6, from 714.10 ± 1580.23 pg/mL to 2423.23 ± 2323.79 pg/mL, p=0.06, **Figure 4b**).

**Table 3:** Changes in serum sCD83levels before and after initiation of various treatments.

Drug	Before treatment	After treatment	p value
IVIG (n=12)	3650.21 ± 6523.94	9937.99 ± 11710.02	0.002
Methotrexate (n=3)	228.40 ± 386.10	806.50 ± 1285.33	0.25
Cladribine (n=10)	3501.17 ± 3552.29	2434.79 ± 2687.63	0.11
Natalizumab (n=7)	3560.05 ± 3682.23	4127.45 ± 3863.22	0.22
Ocrelizumab (n=10)	2622.14 ± 5241.37	1602.32 ± 3259.47	0.16
Rituximab (n=9)	99.97 ± 120.01	91.19 ± 72.86	0.99
Azathioprine (n=6)	714.10 ± 1580.23	2423.23 ± 2323.79	0.06

IVIG, intravenous immunoglobulin.

#### 3.2.2 Immunosuppressive medications increase sCD83 levels secreted by PBMCs

To evaluate the impact of various treatments on sCD83 secretion, PBMCs from HCs were incubated with 20 different drugs commonly used for MS, MOGAD, and NMOSD. A summary of the results for all tested drugs is provided in **Table 4**.

**Table 4:** sCD83 secretion levels in PBMC media: comparing treated versus untreated conditions across 19 treatments.

Drug name	Number of pairs	Baseline result	Treatment result	P-Value
Azathioprine	6	151.20 ± 98.27	210.80 ± 75.73	0.01
Baclofen	5	86.02 ± 55.23	120.30 ± 34.24	0.03
Dimethyl fumarate	4	90.89 ± 33.69	0.00 ± 0.00	0.01
Diroximel fumarate	4	79.08 ± 29.13	0	0.01
Eculizumab	4	120.70 ± 110.70	16.97 ± 27.15	0.09
Fingolimod	5	89.45 ± 55.79	117.60 ± 60.30	0.0004
Inebilizumab	6	189 ± 92.17	78.25 ± 45.54	0.01
IVIG	8	99.93 ± 81.70	123.50 ± 82.49	0.006
Methotrexate	9	105.10 ± 64.19	173.50 ± 62.12	0.0008
Prednisone	4	127.10 ± 107.40	5.70 ± 5.473	0.12
Mycophenolate mofetil	9	104.80 ± 64.42	234.70 ± 93.64	0.001
Natalizumab	3	73.85 ± 43.60	73.67 ± 47.98	0.97
Ocrelizumab	3	84.54 ± 38.22	36.55 ± 5.923	0.19
Rituximab	7	82.12 ± 29.16	110.60 ± 64.61	0.11
Satralizumab	6	189 ± 92.17	158.3 ± 77.13	0.09
Teriflunomide	6	79.00 ± 34.45	68.44 ± 38.16	0.19
Vitamin B12	4	228.20 ± 65.26	161.10 ± 50.33	0.1
Vitamin C	5	214 ± 31.37	238.8 ± 67.97	0.44
Vitamin D	4	83.00 ± 25.65	22.41 ± 22.25	0.0005

IVIG, intravenous immunoglobulin.

**Figure 5:**
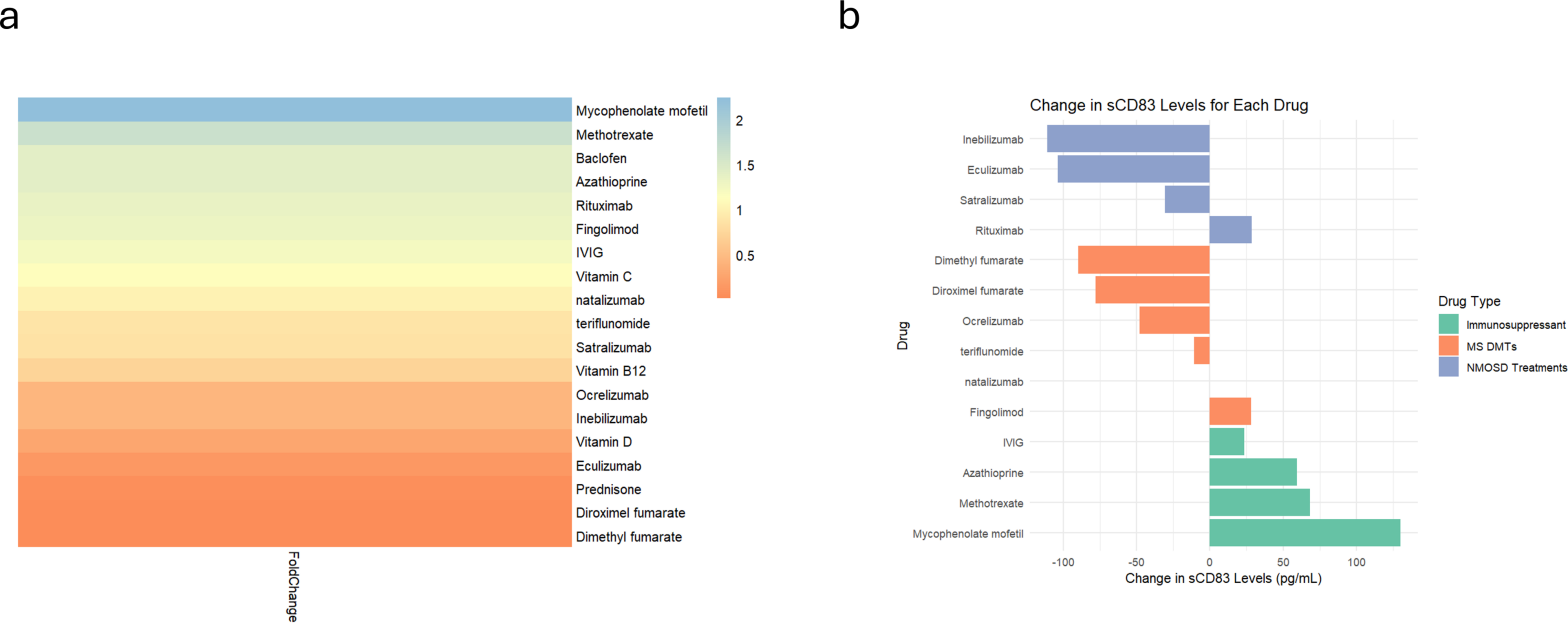
Differential sCD83 secretion in PBMCs responding to different treatments. Analysis of sCD83 secretion in response to 20 different treatments. **(a)** Heatmap illustrating the fold change in sCD83 secretion in PBMCs after receipt of different treatments. **(b)** Analysis of sCD83 secretion in response to MS Disease-Modifying therapies (dimethyl fumarate, diroximel fumarate, fingolimod, natalizumab, ocrelizumab and teriflunomide), NMOSD treatments (eculizumab, inebilizumab, rituximab and teriflunomide), and immunosuppressors (azathioprine, methotrexate, mycophenolate mofetil and IVIG). NMOSD, neuromyelitis optica spectrum disorders; MS, multiple sclerosis; sCD83, soluble CD83; IVIG, intravenous immunoglobulin; PBMCs, Peripheral blood mononuclear cells.

We found that the immunosuppressive medications, MMF (n=9, from 104.80 ± 64.42 pg/mL to 234.70 ± 93.64 pg/mL, p=0.001), methotrexate (n=9, from 105.10 ± 64.19 pg/mL to 173.50 ± 62.12 pg/mL, p=0.008), and AZA (n=9, from 151.20 ± 98.27 pg/mL to 210.80 ± 75.73 pg/mL, p=0.01) significantly upregulate sCD83 secretion (**Figure 5a**), In contrast diroximel fumarate (n=4, from 79.08 ± 29.13 pg/mL to 0 ± 0 pg/mL), dimethyl Fumarate (n=4, from 79.08 ± 29.13 pg/mL to 0 ± 0 pg/mL), methyl prednisone (n=4, from 127.10 ± 107.40 pg/mL to 5.70 ± 5.47 pg/mL) and eculizumab (n=4, from 120.70 ± 110.70 pg/mL to 16.97 ± 27.15 pg/mL) downregulated sCD83 secretion from PBMCs.

Categorizing the treatments that modulate immune responses into three groups—MS disease-modifying therapies (DMTs), NMOSD treatments (anti-IL-6 receptor, B cell depletion, and anti-complement therapies), and immunosuppressants—revealed a significant difference in sCD83 secretion (ANOVA; p = 0.01). Immunosuppressive drugs significantly increased sCD83 secretion compared to NMOSD treatments (70.37 ± 44.17 pg/mL vs. -54.11 ± 65.93 pg/mL, p = 0.02, **Figure 5b**) and DMTs (70.37 ± 44.17 pg/mL vs. -33.09 ± 46.50 pg/mL, p = 0.03, **Figure 5b**). No significant difference was observed between NMOSD treatments and DMTs (p = 0.81).

To determine whether this effect also occurs in patient-derived PBMCs, we incubated PBMCs from MOGAD (n=8) and NMOSD (n=4) patients with IVIG (MOGAD; 204.21 ± 66.39 pg/mL vs 226.27 ± 70.60 pg/mL, p=0.0003 | NMOSD; 133.93 ± 35.32 pg/mL vs 149.02 ± 38.73 pg/mL, p=0.0003), MMF (MOGAD; 198.03 ± 69.18 pg/mL vs 431.47 ± 66.83 pg/mL, p=0.0001 | NMOSD; 133.93 ± 35.32 pg/mL vs 290.58 ± 86.09 pg/mL, p=0.0033), and AZA (MOGAD; 198.03 ± 69.18 pg/mL vs 238.28 ± 54.98 pg/mL, p=0.0009 | NMOSD; 133.93 ± 35.32 pg/mL vs 161.51 ± 34.94 pg/mL, p=0.002). A significant upregulation of sCD83 was observed *in vitro* following treatment with these drugs in all groups **Supplementary Figures S2a–c**.

Moreover, when comparing MMF and AZA, we found that MMF significantly increased sCD83 secretion from PBMCs compared to AZA in MOGAD and NMOSD patients (205.50 ± 79.36 pg/mL vs. 35.65 ± 15.42 pg/mL, p < 0.0001). Furthermore, a similar trend was observed in HCs (129.90 ± 80.11 pg/mL vs. 59.62 ± 39.49 pg/mL, p = 0.06).

## 4 Discussion

Our study reveals significant differences in sCD83 expression among NMOSD, MS, and MOGAD patients, with notably lower levels in NMOSD patients. Moreover, in patients with MOGAD, sCD83 levels were significantly reduced in relapsing compared to monophasic patients and were significantly correlated with higher brain volume. Furthermore, sCD83 levels increase in the sera of patients following treatment with either IVIG or AZA, both effective in preventing relapses, particularly in MOGAD. In culture, the immunosuppressive medications MMF and AZA significantly enhance sCD83 secretion by PBMCs, indicating their potential role in achieving tolerance in these diseases.

Beyond their distinct pathogenic mechanisms, the major demyelinating diseases of the CNS also differ significantly in terms of severity, patterns of disability progression, and clinical outcomes. Most MS patients begin with a relapsing disease course that often transitions to progressive disability over time (46). In contrast, NMOSD and MOGAD are predominantly relapsing diseases, where neurological disability is directly linked to relapses (47). MOGAD generally exhibits a favorable response to immunotherapy, has a better functional prognosis and MRI T2-lesions resolve more often compared to NMOSD patients (48).

We show that sCD83 levels are consistently lower in more severe forms of demyelinating diseases, particularly in NMOSD. NMOSD patients in our cohort exhibit significantly lower sCD83 levels in the serum compared to MS patients and significantly lower serum and CSF levels compared to MOGAD patients. Within the MOGAD group, relapsing patients had significantly lower sCD83 levels than those with a milder, monophasic disease course. These findings suggest that reduced sCD83 levels may be associated with a more severe disease course specifically in antibody-mediated CNS demyelinating disorders. Karampoor et al. observed an increase in sCD83 levels in MS patients, which correlated negatively with disease severity (49). Our data demonstrate that MS patients exhibited lower sCD83 levels compared to HCs, although this reduction was less pronounced than was observed in NMOSD and relapsing MOGAD patients. A possible explanation for this difference is that the MS cohort in their study consisted of treated patients, which could influence sCD83 levels. As in the Karampoor et al. observation, we also find a negative correlation between sCD83 levels and disease severity, a correlation that was more significant in the NMOSD and MOGAD patients. Together, these findings support the hypothesis that sCD83 may play a more significant role in the severity of antibody-mediated demyelinating diseases of the CNS than in MS.

Monitoring sCD83 levels may be particularly valuable in patients initiating a new treatment or those experiencing an early relapse following MOGAD onset, as well as in NMOSD patients. In these individuals, a fast-responding biomarker could be critical, as relapses may lead to irreversible neurological damage (50). Early identification of treatment failure or the timely initiation of therapy in untreated patients could significantly improve clinical outcomes. Accurate identification of treatment failure necessitates careful consideration of the timing of blood collection, as sCD83 levels are influenced by ongoing immunosuppressive therapies. Elevated levels in patients already receiving immunosuppressive therapy may confound interpretation, as a lack of further increase in sCD83 levels in subsequent sample may not reliably indicate relapse risk and could lead to a false negative-response assessment.

We observed a negative correlation between sCD83 serum concentrations and disease severity in both MOGAD and NMOSD conditions. Specifically, within the MOGAD group, sCD83 levels were significantly reduced in relapsing compared to monophasic patients, and there was a positive correlation between sCD83 levels and normalized brain volume, indicating that higher sCD83 may contribute to less severe disease manifestations. Although no correlation was seen between total brain volume and sCD83 levels b NMOSD, there was an inverse correlation between sCD83 levels and VEP scores. These findings collectively indicate that higher levels of sCD83 may exert a protective effect and can serve as a biomarker of disease severity in both disorders.

The underlying reasons why certain MOGAD patients exhibit a monophasic course or experience extended intervals between relapses, as opposed to others who suffer from recurrent disease episodes, remain poorly understood (38). Currently, despite ongoing research, it is not feasible to predict the course of MOGAD, including whether it will be monophasic or relapsing, the timing of potential relapses, and the long-term outcomes (38)

Our findings indicate that at disease onset, higher serum levels of sCD83 in MOGAD patients may provide protection against further relapses. In our data, a serum sCD83 concentration of 706.85 pg/mL could predict a relapsing disease course with high specificity (95.24%), although the sensitivity is relatively low (55.56%). Additional validation studies are necessary to determine the use of sCD83 as a prognostic biomarker and to find the optimal threshold for distinguishing between monophasic and relapsing forms of MOGAD.

In our cohort, we observed a slight, non-significant, increase in serum sCD83 levels during relapses compared to remission in MOGAD, NMOSD, and MS patients. A plausible explanation for this trend lies in the transient nature of CD83 upregulation following exposure to pro-inflammatory stimuli (51). Previous studies have shown that LPS and zymosan rapidly induce CD83 surface expression within the first few hours, followed by a return to baseline levels (51). Similarly, cytokines like TNF-α and IFN-γ trigger a short-term increase in CD83 expression, peaking within a few hours (51). Given the known elevation of these cytokines during relapses (52, 53), it is conceivable that CD83 expression and subsequent shedding into the circulation are occurring temporarily *in vivo*. However, the transient kinetics observed *in vitro* may explain why we detected only modest differences in sCD83 levels, particularly if serum samples were collected at variable time points relative to the onset of relapse. To fully elucidate the temporal dynamics and clinical relevance of CD83 regulation during disease activity, larger longitudinal studies with standardized sampling protocols are needed.

In patients with NMOSD, we found a significant elevation in the expression ratio of mCD83 to sCD83, indicating predominant expression of mCD83 over its soluble counterpart. This imbalance suggests a dysregulation in the alternative splicing mechanisms responsible for CD83 expression, which may contribute to the inflammatory processes at the core of NMOSD pathogenesis. The skewed mCD83/sCD83 ratio could exacerbate pro-inflammatory responses, potentially aggravating the disease state.

While FDA-approved treatments for NMOSD have significantly improved outcomes, a subset of patients may continue to experience relapses or ongoing symptoms (6, 8, 10). Our study found that the current monoclonal antibody treatments used for NMOSD do not lead to an increase in sCD83 levels. In contrast to monoclonal antibody therapies, broader immunosuppressive treatments such as IVIG, AZA, and MMF significantly upregulate sCD83 levels. These agents are known to broadly impact the Treg–DC axis (54, 55). AZA has been shown to impair DC maturation and function, promoting a shift toward a less immunogenic and more tolerogenic phenotype (56). IVIG enhances both the expansion and suppressive function of Tregs and modulates DC activation through downregulation of costimulatory molecules (57, 58). MMF has similarly been reported to inhibit DC maturation and pro-inflammatory cytokine production (59). It is possible that the broad immunomodulatory effects of these treatments on the Treg–DC axis contribute to the observed upregulation of sCD83. Alternatively, sCD83 upregulation itself may be a downstream effector mediating some of the therapeutic benefits of these agents, particularly through its known role in promoting immune tolerance (19, 20). Interestingly, the marked upregulation of sCD83 observed following treatment with immunosuppressive agents may not solely reflect a disease-specific therapeutic mechanism. Instead, it is possible that this increase represents a broader compensatory or rebound response to immune suppression. This interpretation is supported by our *in vitro* findings showing that PBMCs from HCs exhibited a similar pattern of sCD83 elevation when exposed to these agents. In patients with NMOSD who do not respond to FDA-approved monoclonal antibody treatments, measuring sCD83 levels before and after therapy may help guide the choice of alternative treatment. If sCD83 levels are low, broad-spectrum immunosuppressive therapy— either alone or in combination with monoclonal antibody treatments—may represent an appropriate therapeutic approach

It is known that MS treatments like natalizumab and dimethyl fumarate, are not only ineffective for preventing NMOSD relapses but can exacerbate disease severity (60, 61). Notably, we found that these MS treatments downregulate or do not affect sCD83, which may explain their lack of efficacy in NMOSD. While both MMF and AZA are effective for NMOSD treatment, MMF has shown better tolerability and a lower incidence of adverse events. Additionally, some studies indicate that MMF may be associated with a lower relapse risk compared to AZA (62). Notably, our *in vitro* experiments revealed that MMF significantly increased sCD83 secretion from PBMCs in both patients and HCs compared to AZA.

Currently, there are no FDA-approved treatments for MOGAD (11). Commonly used therapies to prevent MOGAD relapses include immunosuppressants such as AZA, MMF and IVIG (11). We observed increased sCD83 levels in both patients and culture systems with these treatments. Although the precise mechanisms underlying IVIG’s therapeutic effects are not fully understood, it is believed to modulate the immune system through multiple pathways (63). Our findings may provide valuable insights into how IVIG may benefit patients with autoimmune diseases.

Limitations of this study include the relatively small number of participants, which can be attributed to the rarity of these disorders. Another limitation is that different treatments can affect sCD83 levels in varying ways; therefore, serum samples intended for disease monitoring should be collected prior to the initiation of therapy. Further research with larger cohorts is necessary to validate the use of sCD83 as a biomarker for CNS antibody-mediated demyelinating disease severity and as a potential therapeutic target for these disorders.

In conclusion, our study consistently identified lower sCD83 levels in NMOSD patients compared to those with MOGAD and HCs. In NMOSD, reduced sCD83 levels correlated with worse VEP scores, while in MOGAD, they were associated with a relapsing disease course and decreased brain volumes. Furthermore, we observed that sCD83 levels increase in the sera of patients following treatment with immunosuppressives or IVIG. These findings highlight sCD83 as a promising biomarker for assessing disease severity, treatment response and early signs of disease activation. Given the risk of irreversible neurological damage associated with relapses, a rapidly responsive biomarker like sCD83 could aid timely therapeutic decision-making. Collectively, our results support sCD83 as both a therapeutic target and a biomarker in CNS demyelinating diseases.

## Data Availability

The raw data supporting the conclusions of this article will be made available by the authors, without undue reservation.

## Supplementary material

Supplementary Figure 1 sCD83 levels are slightly upregulated during relapse. sCD83 levels during relapses and remission. **(a)** sCD83 levels are slightly higher during relapses in MOGAD patients (n=19; 4940 ± 6155 vs 6803 ± 8977, p=0.18) **(b)** sCD83 levels during relapses and remission. **(a)** sCD83 levels are slightly higher during relapses in MS patients (n=6; 3427 ± 5236 vs 5547 ± 6779, p=0.53) **(c)** sCD83 levels are slightly higher during relapses in NMOSD patients (n=7; 88.86 ± 185.50 vs 348.30 ± 779.80, p=0.29) MOGAD, myelin oligodendrocyte glycoprotein antibody-associated disease; NMOSD, neuromyelitis optica spectrum disorders; MS, multiple sclerosis; ONNIDs, other non-inflammatory neurological disorders; sCD83, soluble CD83

Supplementary Figure 2 sCD83 levels are similar at remission and relapsing patients. sCD83 levels of remission and relapsing patients **(a)** MOGAD patients during remission and relapse (7119.32 ± 10232.09 vs 6913.49 ± 8186.14, p=0.93) **(b)** NMOSD patients during remission and relapse (1066.75 ± 1831.68 vs 1183.35 ± 2292.41, p=0.84) **(c)** MS patients during remission and relapse (5989.19 ± 9332.90 vs 5590.15 ± 7508.02, p=0.87). MOGAD, myelin oligodendrocyte glycoprotein antibody-associated disease; NMOSD, neuromyelitis optica spectrum disorders; MS, multiple sclerosis; sCD83, soluble CD83

Supplementary Figure 3 IVIG, AZA, and MMF increased sCD83 Secretion from PBMCs in both patients and HCs. Effects of various treatments on sCD83 secretion in PBMCs from HCs, MOGAD and NMOSD patients. **(a)** IVIG (99.9 ± 81.70 pg/mL vs 123.50 ± 82.49 pg/mL, p=0.006), azathioprine (151.20 ± 98.27 pg/mL vs 210.80 ± 75.73 pg/mL, p=0.01) and mycophenolate mofetil (104.80 ± 64.42 pg/mL vs 234.70 ± 9.64 pg/mL, p=0.001) increase sCD83 secretion by PBMCs of HCs. **(b)** IVIG (204.21 ± 66.39 pg/mL vs 226.27 ± 70.60 pg/mL, p=0.0003), azathioprine (198.03 ± 69.18 pg/mL vs 238.28 ± 54.98 pg/mL, p=0.0009) and mycophenolate mofetil (198.03 ± 69.18 pg/mL vs 431.47 ± 66.83 pg/mL, p=0.0001) increase sCD83 secretion by PBMCs of MOGAD patients. **(c)** IVIG (133.93 ± 35.32 pg/mL vs 149.02 ± 38.73 pg/mL, p=0.0003), azathioprine (133.93 ± 35.32 pg/mL vs 161.51 ± 34.94 pg/mL, p=0.002) and mycophenolate mofetil (133.93 ± 35.32 pg/mL vs 290.58 ± 86.09 pg/mL, p=0.003) increase sCD83 secretion by PBMCs of NMOSD patients. HC, healthy control; MOGAD, myelin oligodendrocyte glycoprotein antibody-associated disease; NMOSD, neuromyelitis optica spectrum disorders; sCD83, soluble CD83; IVIG, intravenous immunoglobulin; PBMCs, Peripheral blood mononuclear cells.
