# Supplementary figures and images for: Reduced tolerogenic factor sCD83 in NMOSD and relapsing MOGAD: a potential new therapeutic pathway
Source: Front Immunol. 2025 Jul 24;16:1620069. doi: 10.3389/fimmu.2025.1620069 (PMC12328149; doi:10.3389/fimmu.2025.1620069)

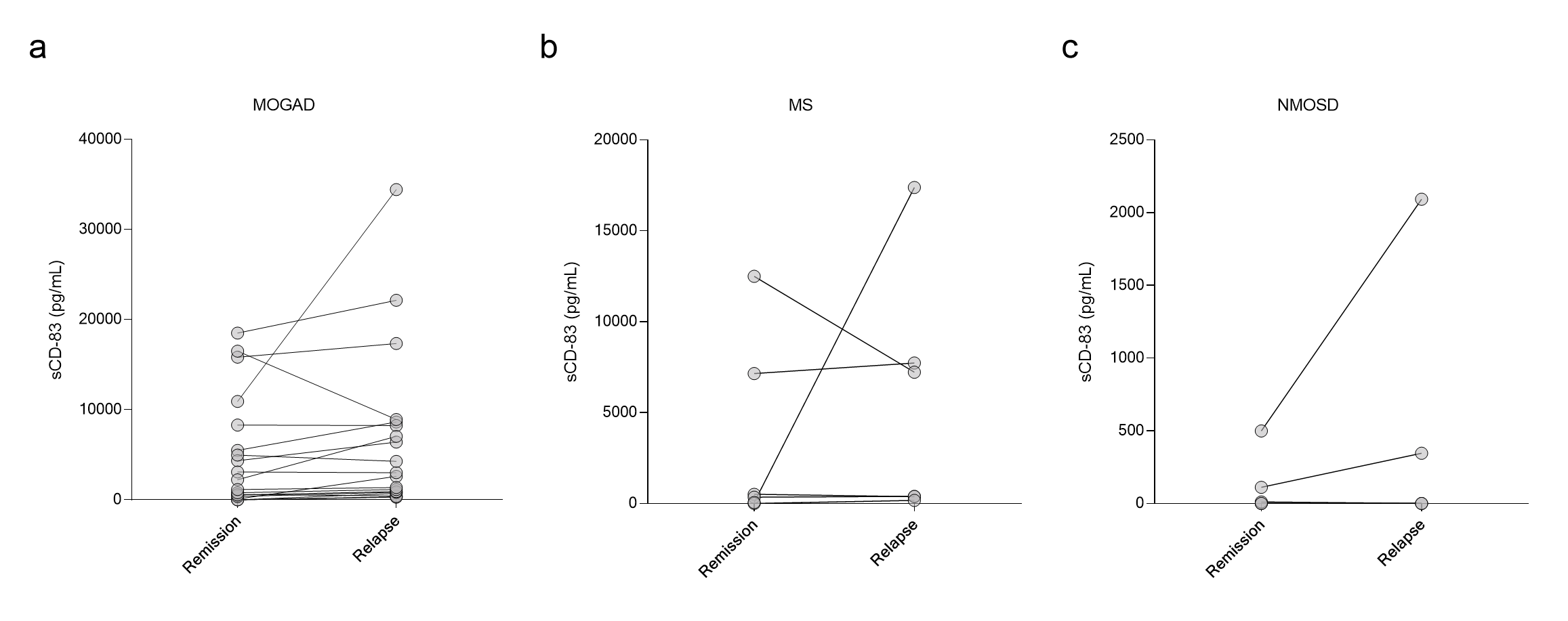

Supplement: Supplementary Figure 1 — sCD83 levels are slightly upregulated during relapse. sCD83 levels during relapses and remission. (a) sCD83 levels are slightly higher during relapses in MOGAD patients (n=19; 4940 ± 6155 vs 6803 ± 8977, p=0.18) (b) sCD83 levels during relapses and remission. (a) sCD83 levels are slightly higher during relapses in MS patients (n=6; 3427 ± 5236 vs 5547 ± 6779, p=0.53) (c) sCD83 levels are slightly higher during relapses in NMOSD patients (n=7; 88.86 ± 185.50 vs 348.30 ± 779.80, p=0.29) MOGAD, myelin oligodendrocyte glycoprotein antibody-associated disease; NMOSD, neuromyelitis optica spectrum disorders; MS, multiple sclerosis; ONNIDs, other non-inflammatory neurological disorders; sCD83, soluble CD83 [file Image1.tif]

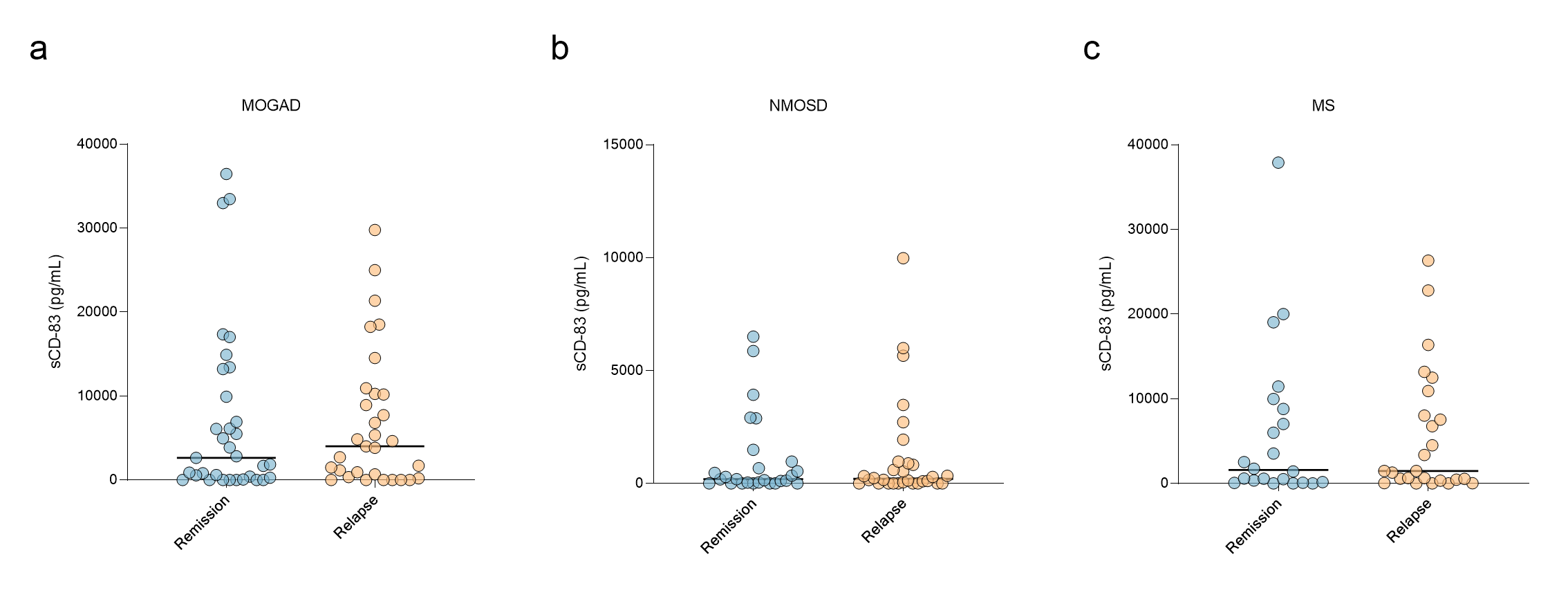

Supplement: Supplementary Figure 2 — sCD83 levels are similar at remission and relapsing patients. sCD83 levels of remission and relapsing patients (a) MOGAD patients during remission and relapse (7119.32 ± 10232.09 vs 6913.49 ± 8186.14, p=0.93) (b) NMOSD patients during remission and relapse (1066.75 ± 1831.68 vs 1183.35 ± 2292.41, p=0.84) (c) MS patients during remission and relapse (5989.19 ± 9332.90 vs 5590.15 ± 7508.02, p=0.87). MOGAD, myelin oligodendrocyte glycoprotein antibody-associated disease; NMOSD, neuromyelitis optica spectrum disorders; MS, multiple sclerosis; sCD83, soluble CD83 [file Image2.tif]

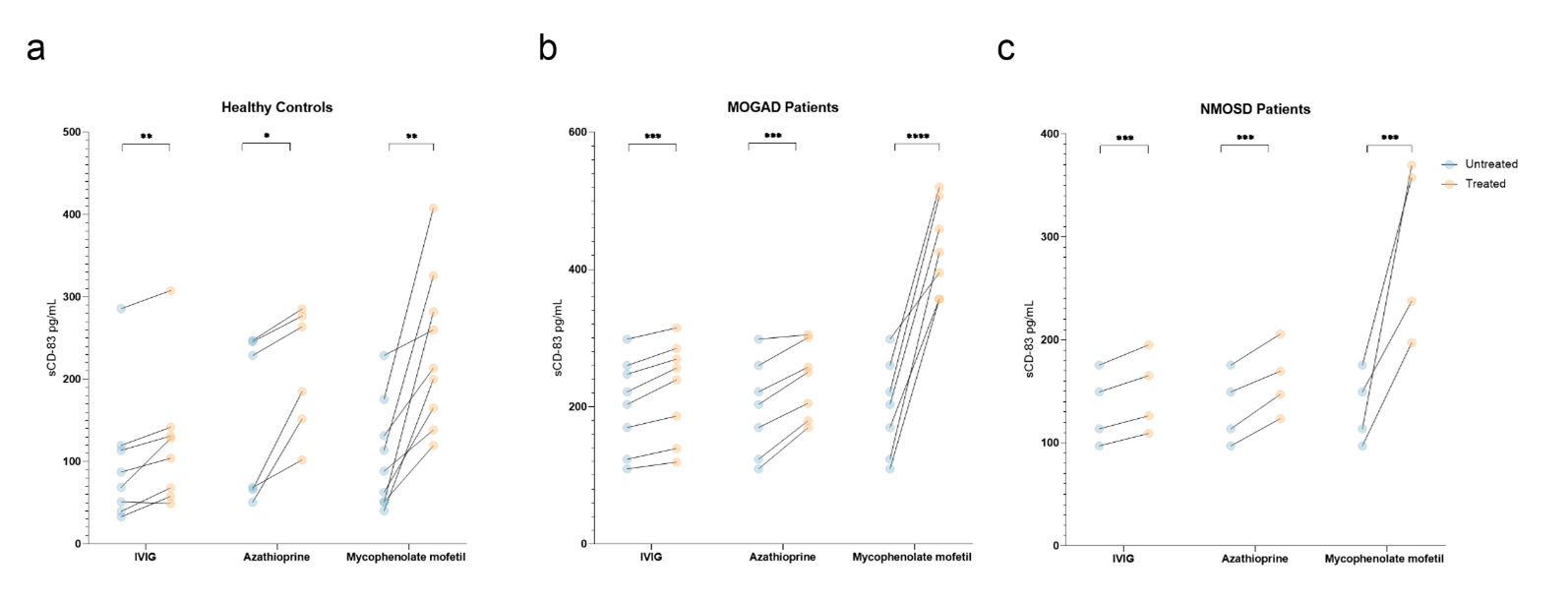

Supplement: Supplementary Figure 3 — IVIG, AZA, and MMF increased sCD83 Secretion from PBMCs in both patients and HCs. Effects of various treatments on sCD83 secretion in PBMCs from HCs, MOGAD and NMOSD patients. (a) IVIG (99.9 ± 81.70 pg/mL vs 123.50 ± 82.49 pg/mL, p=0.006), azathioprine (151.20 ± 98.27 pg/mL vs 210.80 ± 75.73 pg/mL, p=0.01) and mycophenolate mofetil (104.80 ± 64.42 pg/mL vs 234.70 ± 9.64 pg/mL, p=0.001) increase sCD83 secretion by PBMCs of HCs. (b) IVIG (204.21 ± 66.39 pg/mL vs 226.27 ± 70.60 pg/mL, p=0.0003), azathioprine (198.03 ± 69.18 pg/mL vs 238.28 ± 54.98 pg/mL, p=0.0009) and mycophenolate mofetil (198.03 ± 69.18 pg/mL vs 431.47 ± 66.83 pg/mL, p=0.0001) increase sCD83 secretion by PBMCs of MOGAD patients. (c) IVIG (133.93 ± 35.32 pg/mL vs 149.02 ± 38.73 pg/mL, p=0.0003), azathioprine (133.93 ± 35.32 pg/mL vs 161.51 ± 34.94 pg/mL, p=0.002) and mycophenolate mofetil (133.93 ± 35.32 pg/mL vs 290.58 ± 86.09 pg/mL, p=0.003) increase sCD83 secretion by PBMCs of NMOSD patients. HC, healthy control; MOGAD, myelin oligodendrocyte glycoprotein antibody-associated disease; NMOSD, neuromyelitis optica spectrum disorders; sCD83, soluble CD83; IVIG, intravenous immunoglobulin; PBMCs, Peripheral blood mononuclear cells. [file Image3.tif]
