# Supplementary material for: Reduced tolerogenic factor sCD83 in NMOSD and relapsing MOGAD: a potential new therapeutic pathway
Source: Front Immunol. 2025 Jul 24;16:1620069. doi: 10.3389/fimmu.2025.1620069 (PMC12328149; doi:10.3389/fimmu.2025.1620069)
Supplement: Supplementary file 4 [file Table1.docx]

**Supplementary Table 1**: Drug Concentrations Applied to PBMCs Cultures

| Name | Concentration |
| --- | --- |
| Azathioprine(1) | 500 ug/mL |
| Baclofen | 10 uM |
| Dimethyl fumarate(2) | 25uM |
| Diroximel fumarate(2) | 25uM |
| Eculizumab(3) | 2 ug/mL |
| Fingolimod(4) | 5 uM |
| Inebilizumab(5) | 10 μg/ml |
| IVIG(6) | 10mg/mL |
| Methotrexate(7) | 1 uM |
| Methyl Prednisone(8) | 100 ug/mL |
| Mycophenolate mofetil(9) | 1 uM |
| natalizumab | 100 μg/mL |
| Ocrelizumab(10) | 3ug/Ml |
| Rituximab(10) | 3ug/Ml |
| Satralizumab(11) | 100 ug/mL |
| teriflunomide(12) | 50 μM |
| Vitamin b12(13) | 10 ug/mL |
| Vitamin C(14) | 1 mg/mL |
| Vitamin D(15) | 0.1 uM |

**References:**

1. Homo-Delarche F, Bach JF, Dardenne M. In vitro inhibition of prostaglandin production by azathioprine and 6-merchaptopurine. *Prostaglandins* (1988) 35:479–491. doi: 10.1016/0090-6980(88)90024-X

2. Guerriero C, Puliatti G, Di Marino T, Tata AM. Effects Mediated by Dimethyl Fumarate on In Vitro Oligodendrocytes: Implications in Multiple Sclerosis. *Int J Mol Sci* (2022) 23:3615. doi: 10.3390/ijms23073615

3. Harder MJ, Höchsmann B, Dopler A, Anliker M, Weinstock C, Skerra A, Simmet T, Schrezenmeier H, Schmidt CQ. Different Levels of Incomplete Terminal Pathway Inhibition by Eculizumab and the Clinical Response of PNH Patients. *Front Immunol* (2019) 10:1639. doi: 10.3389/fimmu.2019.01639

4. Colombo E, Bassani C, De Angelis A, Ruffini F, Ottoboni L, Comi G, Martino G, Farina C. Siponimod (BAF312) Activates Nrf2 While Hampering NFκB in Human Astrocytes, and Protects From Astrocyte-Induced Neurodegeneration. *Front Immunol* (2020) 11:635. doi: 10.3389/fimmu.2020.00635

5. Herbst R, Wang Y, Gallagher S, Mittereder N, Kuta E, Damschroder M, Woods R, Rowe DC, Cheng L, Cook K, et al. B-Cell Depletion In Vitro and In Vivo with an Afucosylated Anti-CD19 Antibody. *J Pharmacol Exp Ther* (2010) 335:213–222. doi: 10.1124/jpet.110.168062

6. Sticherling M, Trawinski H. Effects of Intravenous Immunoglobulins on Peripheral Blood Mononuclear Cell Activation *in Vitro*. *Ann N Y Acad Sci* (2007) 1110:694–708. doi: 10.1196/annals.1423.072

7. Nesher G, Moore TL. The in vitro effects of methotrexate on peripheral blood mononuclear cells: Modulation by methyl donors and spermidine. *Arthritis Rheum* (1990) 33:954–959. doi: 10.1002/art.1780330706

8. Gelati M. Methylprednisolone Acts on Peripheral Blood Mononuclear Cells and Endothelium in Inhibiting Migration Phenomena in Patients With Multiple Sclerosis. *Arch Neurol* (2002) 59:774. doi: 10.1001/archneur.59.5.774

9. Heinschink A, Raab M, Daxecker H, Griesmacher A, Müller MM. In vitro effects of mycophenolic acid on cell cycle and activation of human lymphocytes. *Clin Chim Acta* (2000) 300:23–28. doi: 10.1016/S0009-8981(00)00297-7

10. Ito S, Miwa K, Hattori C, Aida T, Tsuchiya Y, Mori K. Highly sensitive *in vitro* cytokine release assay incorporating high-density preculture. *J Immunotoxicol* (2021) 18:136–143. doi: 10.1080/1547691X.2021.1984617

11. Takeshita Y, Fujikawa S, Serizawa K, Fujisawa M, Matsuo K, Nemoto J, Shimizu F, Sano Y, Tomizawa-Shinohara H, Miyake S, et al. IL-6 blockade suppresses the blood-brain barrier disorder, leading to prevention of onset of NMOSD. (2021) doi: 10.1101/2021.01.28.428564

12. Wostradowski T, Prajeeth CK, Gudi V, Kronenberg J, Witte S, Brieskorn M, Stangel M. In vitro evaluation of physiologically relevant concentrations of teriflunomide on activation and proliferation of primary rodent microglia. *J Neuroinflammation* (2016) 13:250. doi: 10.1186/s12974-016-0715-3

13. Sakane T, Takada S, Kotani H, Tsunematsu T. Effects of methyl-b12 on thein vitro immune functions of human T lymphocytes. *J Clin Immunol* (1982) 2:101–109. doi: 10.1007/BF00916893

14. Wang G, Yin T, Wang Y. In vitro and in vivo assessment of high-dose vitamin C against murine tumors. *Exp Ther Med* (2016) 12:3058–3062. doi: 10.3892/etm.2016.3707

15. Díaz L, Noyola-Martínez N, Barrera D, Hernández G, Avila E, Halhali A, Larrea F. Calcitriol inhibits TNF-α-induced inflammatory cytokines in human trophoblasts. *J Reprod Immunol* (2009) 81:17–24. doi: 10.1016/j.jri.2009.02.005
